# Platinum Rechallenge in Platinum-Resistant Ovarian Cancer: Clinical Outcomes and the Impact of BRCA Status

**DOI:** 10.3390/cancers18132062

**Published:** 2026-06-25

**Authors:** David García-Illescas, Víctor Navarro, Lorena Fariñas-Madrid, Carmen García-Durán, Juan Francisco Grau Béjar, Lucia Musacchio, Roberta Mazzeo, Irene Giannubilo, Guillermo Villacampa, Ana Oaknin

**Affiliations:** 1 Gynaecologic Cancer Programme, Medical Oncology Department, Vall d’Hebron Institute of Oncology (VHIO), Hospital Universitari Vall d’Hebron, Vall d’Hebron Barcelona Hospital Campus, 08035 Barcelona, Spain; 2 PhD Programme in Medicine, Department of Medicine, Universitat Autònoma de Barcelona (UAB), 08193 Bellaterra, Spain; 3 Statistics Unit, Vall d’Hebron Institute of Oncology (VHIO), 08035 Barcelona, Spain; 4 Gynecological Cancer Translational Research Laboratory, INSERM UMR 981, Institut Gustave Roussy, Université Paris-Saclay, 94805 Villejuif, France; 5 Department of Woman, Child and Public Health, Fondazione Policlinico Universitario A. Gemelli IRCCS, 00168 Rome, Italy

**Keywords:** platinum rechallenge, platinum-resistant ovarian cancer, BRCA mutation, treatment sequencing, PARP inhibitors

## Abstract

**Objective**: To evaluate the clinical benefit of platinum rechallenge in patients with platinum-resistant ovarian cancer (PROC) and to explore the predictive role of *BRCA* mutation status. **Methods**: We retrospectively evaluated platinum rechallenge in PROC at Vall d’Hebron University Hospital between 2010 and 2023. Eligibility required ≥1 prior non-platinum regimen and ≥12 months since the last platinum dose. Objective response rate (ORR) per RECIST 1.1 and survival outcomes were analyzed by *BRCA* status and prior treatment lines. **Results**: ORR was 57% (95%CI, 42.1–73.0%) in the overall cohort (*n* = 63), 77% in *BRCA*-mutated patients (*n* = 13), and 50% in BRCA wild-type patients (*n* = 46). Median progression-free survival (PFS) was 7.3 months overall, which was longer in BRCA-mutated versus BRCA wild-type patients (8.4 vs. 7.4 months; HR 0.47, 95%CI 0.23–0.94; *p* = 0.033). An exploratory subgroup analysis suggested longer PFS among *BRCA*-mutated patients with ≤4 prior lines; however, this small subgroup finding should be considered hypothesis-generating. A platinum-free interval >6 months after rechallenging was observed in 38.5% of BRCA-mutated versus 13% of *BRCA* wild-type patients. **Conclusions**: Platinum rechallenge showed clinically meaningful activity in selected patients with PROC after an extended interval of intervening non-platinum therapy. These findings support the concept that platinum sensitivity may be dynamic and suggest that *BRCA* status and clinical selection factors may help inform individualized treatment sequencing in the post-PARP inhibitor era. Prospective validation with broader molecular characterization is warranted.

## Simple Summary

Patients with ovarian cancer whose disease recurs shortly after platinum chemotherapy are usually considered platinum-resistant and are commonly treated with non-platinum drugs. However, some patients may regain sensitivity to platinum after a long interval during which other treatments are given. In this retrospective study, we evaluated 63 patients with platinum-resistant ovarian cancer who later received platinum rechallenge after at least 12 months since their previous platinum treatment. More than half of the patients responded, and outcomes appeared more favorable among patients with *BRCA*-mutated tumors. These findings suggest that platinum resistance may not always be permanent and that *BRCA* status could help identify patients who may still benefit from platinum rechallenge. Prospective studies with broader molecular testing are needed to confirm these results.

## 1. Introduction

Recurrent epithelial ovarian cancer remains a major therapeutic challenge [1].

The traditional classification of recurrent ovarian cancer relies on the treatment-free interval of platinum, with disease recurring within 6 months after the last platinum dose defined as platinum-resistant ovarian cancer [2]. This binary classification has historically guided treatment decisions, with these patients typically receiving non-platinum chemotherapy with or without bevacizumab [3], and more recently with mirvetuximab soravtansine in folate receptor alpha-positive tumors [4]. However, outcomes remain poor, and treatment selection is still guided by limited predictive biomarkers beyond FRα expression [5].

Nevertheless, the traditional binary classification of platinum sensitivity is increasingly being challenged. The Gynecologic Cancer InterGroup emphasized that the platinum-free interval alone should not dictate treatment selection, recognizing that platinum sensitivity may be restored after a sufficient platinum-free interval during which non-platinum therapies are administered [2,6,7]. Similarly, ESMO guidelines acknowledge platinum rechallenge as an option in selected patients with PROC, particularly when the disease has not progressed during prior platinum therapy [8]. Retrospective series have reported response rates of approximately 23–48% with platinum rechallenge after intervening non-platinum therapy [9,10].

The molecular basis for the potential restoration of platinum sensitivity likely involves complex mechanisms. *BRCA*1/2 alterations are biologically relevant in this context because homologous recombination repair deficiency confers increased platinum sensitivity [11,12,13]. However, whether *BRCA* status predicts benefit from platinum rechallenge in the resistant setting remains poorly characterized, as most published data predate the widespread use of poly(ADP-ribose) polymerase (PARP) inhibitors and contemporary targeted therapies [10,14,15,16].

In our clinical practice at a tertiary gynecologic oncology center, we have observed that selected patients with platinum-resistant ovarian cancer who receive non-platinum therapies for extended periods and subsequently undergo platinum rechallenge demonstrate meaningful clinical responses. This question is increasingly relevant in the contemporary treatment landscape, where patients may receive multiple lines of therapy before reconsidering platinum [17]. Furthermore, no studies have evaluated the predictive role of *BRCA* status in this specific clinical scenario, representing a critical knowledge gap for treatment sequencing decisions [9,10].

We therefore conducted this retrospective study to assess the clinical benefit and safety of platinum rechallenge in patients with platinum-resistant ovarian cancer who had received at least 12 months of non-platinum therapy, with particular focus on *BRCA* mutation status as a potential predictive biomarker. Our hypothesis was that platinum sensitivity may represent a dynamic state that can be restored in selected patients.

## 2. Patients and Methods

### 2.1. Study Design and Population

This is a retrospective, observational, and single-center study that included patients with platinum-resistant ovarian cancer who received platinum rechallenge at Vall d’Hebron University Hospital in Barcelona, Spain, between January 2010 and July 2023.

Platinum-resistant ovarian cancer was defined as disease progression occurring within 6 months of the last platinum dose. Eligibility for platinum rechallenge requires at least one prior non-platinum regimen and a minimum interval of 12 months between the last platinum dose and platinum rechallenge. All patients had documented disease progression, either clinical or radiological, prior to platinum rechallenge. The interval between the last platinum dose and disease progression on the most recent non-platinum therapy prior to rechallenge was defined as the treatment-free interval before rechallenging (TFIp). Patients with primary platinum-refractory disease (progression during or within 3 months of first-line platinum-based chemotherapy) were not considered for platinum rechallenge and were not included. The study cohort comprised patients with available radiological response assessment following platinum rechallenge.

The study was conducted in accordance with the ethical guidelines of the Declaration of Helsinki. This observational retrospective study was conducted and reported in accordance with the STROBE guidelines [18].

### 2.2. Clinical Data

Epidemiological, clinical, histological, molecular, and radiological data were obtained from the patients’ electronic medical records. Detailed data on previous therapies and patient outcomes were also collected. The total number of prior treatment lines included both platinum-based and non-platinum therapies administered in platinum-sensitive and platinum-resistant settings. Response evaluation was performed using thorax, abdomen, and pelvic computed tomography (CT) scans in all patients, according to the Response Evaluation Criteria in Solid Tumors (RECIST) version 1.1. RECIST 1.1 response was retrospectively assigned based on available radiological assessments and clinical records. Progression-free survival was defined as the time interval from the start of platinum rechallenge to disease progression or death from any cause. Overall survival was defined as the time from initiation of platinum-rechallenge to death from any cause. The platinum-free interval after rechallenging was defined as the time from the last dose of platinum-based therapy administered during rechallenge to the date of disease progression.

Regarding molecular data, patients were tested for *BRCA*1 and *BRCA*2 germline mutations. Additionally, a somatic targeted-gene, next-generation sequencing (NGS) panel was performed in a subset of patients, either locally (using Amplicon NGS or the VHIO-300 panel) as part of the institutional molecular pre-screening program or through external commercial platforms, depending on availability at the time of testing. The tumor tissue samples analyzed by NGS may correspond to various stages of the disease, including primary disease, platinum-sensitive recurrence, or platinum-resistant recurrence. Any pathogenic variant identified in *BRCA*1 or *BRCA*2, whether germline or somatic, was classified as a *BRCA* mutation. For analytical purposes, patients with unknown *BRCA* status were categorized within the *BRCA* wild-type subgroup. As testing platforms, gene coverage, and tissue timing varied over the study period, non-BRCA molecular alterations were summarized descriptively and were not incorporated into formal efficacy analyses.

Anonymized patient clinical data were collected and managed in the online electronic program REDCap (Research Electronic Data Capture, Vanderbilt University, Nashville, TN, USA) hosted at Vall d’Hebron Institute of Oncology (VHIO). The follow-up data cut-off was May 2024.

### 2.3. Outcomes

The main outcome was objective response rate, according to RECIST 1.1, assessed in the overall population and according to *BRCA* mutation status. Additional outcomes included: (1) progression-free survival per RECIST 1.1 in the overall population and according to *BRCA*1/2 status and number of prior treatment lines; (2) overall survival in the overall population and by *BRCA* status; (3) exploratory analyses of outcomes by clinical categories; (4) platinum-free interval following the platinum rechallenge in the overall population and by *BRCA*1/2 mutation status; and (5) safety of platinum rechallenge, assessed according to Common Terminology Criteria for Adverse Events (CTCAE) version 5.0.

### 2.4. Statistical Analysis

A descriptive analysis of clinical variables was conducted for the overall population, as well as the *BRCA*-mutated and *BRCA* wild-type cohorts. Categorical variables were summarized using frequencies and percentages, while numerical variables were reported as medians with interquartile ranges (IQR). To assess differences in clinical variables between the *BRCA*-mutated and *BRCA* wild-type cohorts, the Wilcoxon test was used for numerical variables, and the chi-square test was applied for categorical variables.

In terms of objective response rate, frequencies and percentages were reported, and a chi-square test was performed to calculate the *p*-value and identify significant differences between groups.

Progression-free and overall survival were analyzed using the Kaplan–Meier method and reported along with the associated 95% confidence interval (95%CI). A Cox proportional hazards model was used to detect differences in survival endpoints; hazard ratio (HR) with 95%CI and *p*-value were reported. Exploratory multivariable Cox proportional hazards models were performed for progression-free survival. Given the limited sample size, models were kept parsimonious and included clinically relevant covariates to reduce the risk of overfitting.

All statistical analyses were performed using R software version 4.2.2. Results were considered statistically significant if *p* < 0.05.

## 3. Results

Between 2010 and 2023, a total of 63 patients with platinum-resistant ovarian cancer underwent platinum rechallenge, and all were evaluable for response. Patients’ demographics, clinical, and molecular characteristics are summarized in Table 1. The median age was 57 years (interquartile range [IQR], 47–63). Most patients (97%) had an Eastern Cooperative Oncology Group (ECOG) performance status of 0 or 1, while 3% had an ECOG of 2. Most patients had a high-grade serous histology. Only two cases were high-grade endometrioid carcinoma, and three had clear cell histology. The median number of prior lines of treatment was 6 (IQR 5–6). Previous treatment lines before platinum rechallenge included both standard-of-care regimens and investigational therapies administered within clinical trials. A total of 29 patients (46%) had received prior PARP inhibitor therapy. The median treatment-free interval of platinum before the platinum rechallenge was 25 months (IQR 20–33). The most used platinum doublet at rechallenge was the combination of pegylated liposomal doxorubicin plus carboplatin (25 patients, 41%), followed by paclitaxel plus carboplatin (24 patients, 39%) and gemcitabine plus carboplatin (12 patients, 20%). A total of 13 patients (20%) harbored a *BRCA* mutation, either germline or somatic: 11 patients had a *BRCA*1 mutation (17%), and two patients had a *BRCA*2 mutation (3%). Additionally, 46 patients (73%) had *BRCA* wild-type tumors, while *BRCA* status was not available for four patients (6%).

Additional molecular profiling beyond *BRCA*1/2 was available for 40 of 63 patients (63.5%). The most frequent alteration was a pathogenic/likely pathogenic variant in TP53, identified in 32 patients (80.0% of profiled patients). Copy-number gain/amplification of CCNE1 was observed in four patients (10.0%). RB1 alterations were observed in three patients (7.5%), including two pathogenic/likely pathogenic variants and one copy-number gain/duplication. PTEN alterations were reported in three patients (7.5%). Alterations in Fanconi pathway genes and NF1/NF2 were infrequent. Given the retrospective nature of the cohort and the heterogeneity of molecular testing platforms, tissue timing, and reporting format, these findings were summarized descriptively and were not incorporated into formal efficacy analyses [Table 2].

The objective response rate was 57.1% in the overall population (95%CI, 42.1–73.0), with 2 patients achieving a complete response and 34 having a partial response. A total of 17 patients achieved stable disease as the best response (27%, 95%CI [16.6–39.7]). Thus, the disease control rate was 84.1% [95%CI, 72.7–92.1]. Disease progression at first evaluation was observed in 10 patients (15.9%, 95%CI [7.9–29.1]). The objective response rate in women with *BRCA*1/2 mutated tumors was 76.9% (95%CI, 46.2–95.0) compared to 50.0% (95%CI, 34.9–65.1) of women in the *BRCA* wild-type subgroup [Table 3].

**Figure 1:**
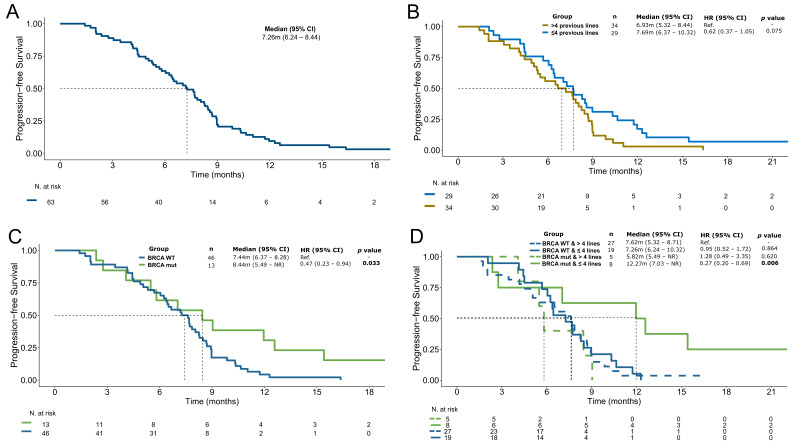
Kaplan–Meier curves for progression-free survival (PFS). (**A**) Entire cohort. (**B**) Stratified by number of prior treatment lines (≤4 vs. >4). (**C**) Stratified by *BRCA* mutation status. (**D**) Combined stratification by *BRCA* status and prior treatment lines. Median PFS and hazard ratios (HR) are shown where applicable. Dashed horizontal and vertical lines denote the 50% survival probability and the corresponding median progression-free survival estimate, respectively.

In the overall cohort, median progression-free survival was 7.3 months (95%CI, 6.2–8.3) [Figure 1A]. According to the number of prior treatment lines, the median progression-free survival for patients who had received four or fewer lines was 7.69 months (95%CI, 6.37–10.32), compared to 6.93 months (95%CI, 5.32–8.94) for those with more than four prior lines (HR 0.62, 95%CI, 0.37–1.05; *p* = 0.075) [Figure 1B].

By *BRCA* status, median progression-free survival was 8.44 months (95%CI, 5.49-NR) in *BRCA*-mutated patients and 7.44 months (95%CI, 6.37–8.28) in the *BRCA* wild-type subgroup (HR 0.47, 95%CI, 0.23–0.94; *p* = 0.033) [Figure 1C].

In an exploratory subgroup analysis, patients with *BRCA*-mutated tumors treated after ≤4 prior lines (*n* = 8) showed numerically longer progression-free survival, with a median of 12.3 months (95%CI, 7.03-NR; HR 0.27, *p* = 0.006) [Figure 1D].

**Figure 2:**
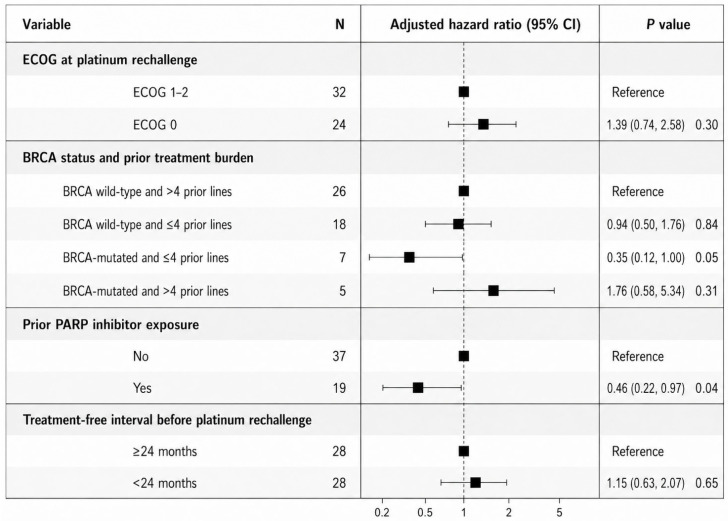
Forest plot showing adjusted hazard ratios for progression-free survival after platinum rechallenge. The multivariable model included ECOG at platinum rechallenge, *BRCA* status, prior treatment burden, prior PARP inhibitor exposure, and treatment-free interval before platinum rechallenge. HR < 1 indicates a lower risk of progression or death.

An exploratory multivariable Cox model for progression-free survival was performed in 56 evaluable patients. The model included ECOG performance status at rechallenge, combined *BRCA*/prior-lines category, prior PARP inhibitor exposure, and treatment-free interval before platinum rechallenge. In this model, *BRCA*-mutated patients treated after ≤4 prior lines showed a numerically lower risk of progression or death compared with *BRCA* wild-type patients treated after >4 prior lines (HR 0.35, 95%CI 0.12–1.00; *p* = 0.05). Prior PARP inhibitor exposure was also associated with longer progression-free survival (HR 0.46, 95%CI 0.22–0.97; *p* = 0.04) [Figure 2].

Median overall survival for the entire cohort was 15.84 months (95%CI, 11.33–19.48). According to the number of prior treatment lines, median overall survival was 19.22 months (95%CI, 14.95–31.93) in patients treated after ≤4 prior lines, compared with 11.33 months (95%CI, 9.56–24.18) in those treated after >4 prior lines (HR 0.82, 95%CI, 0.48–1.42; *p* = 0.477). In the subgroup with *BRCA*1/2-mutated tumors, median overall survival was 31.93 months (95%CI, 10.4–NR), and 15.84 months (95%CI, 11.33–19.48) in *BRCA* wild-type patients (HR 0.63, 95%CI, 0.31–1.28; *p* = 0.204). In the exploratory combined subgroup analysis, patients with *BRCA*1/2 mutations treated after ≤4 prior lines of therapy (*n* = 8) showed a numerically longer overall survival, with a median of 34.90 months (95%CI, 31.93–NR) [Supplementary Figure S1].

In the overall population, the median platinum-free interval after platinum rechallenge was 2.8 months (95%CI, 1.9–3.7). By *BRCA* status, the median platinum-free interval was 2.9 months (95%CI, 0.9-NR) in *BRCA*-mutated patients compared with 2.5 months (95%CI, 1.9–3.7) in the *BRCA* wild-type subgroup (HR 0.55; 95%CI, 0.28–1.19; *p* = 0.085). Eleven patients (17.5%) achieved a platinum-free interval ≥6 months following platinum rechallenge. Of these, five patients carried a *BRCA* mutation (38.5% of the *BRCA*-mutated subgroup), while the remaining six (13.0% of the *BRCA* wild-type subgroup) did not.

**Figure 3:**
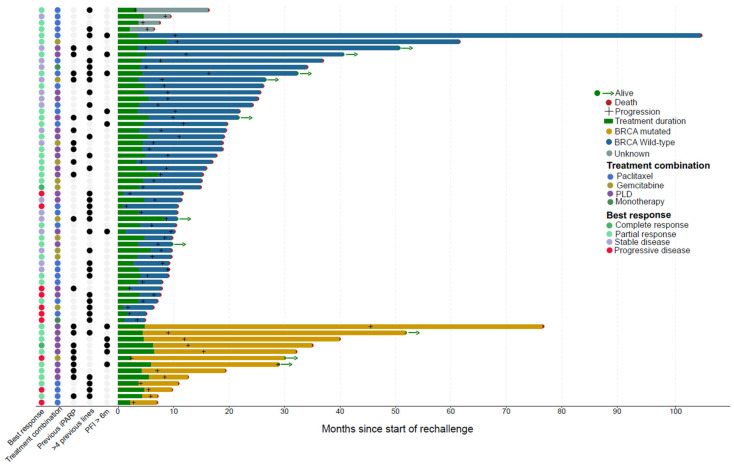
Swimmer plot illustrating treatment duration, response, and survival status for each patient following platinum rechallenge. Bars represent the duration of therapy; symbols indicate the best response and survival status. Colors denote *BRCA* mutation status and treatment regimen.

A swimmer plot illustrates individual patient treatment duration and clinical outcomes, highlighting patterns of response in relation to clinical and molecular characteristics [Figure 3].

**Figure 4:**
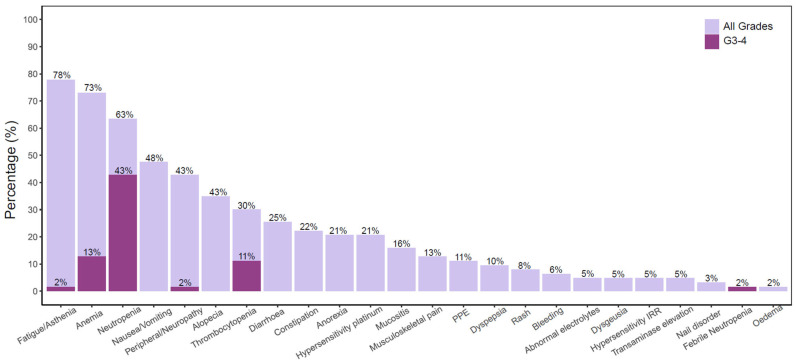
Treatment-related adverse events during platinum rechallenge. Distribution of hematologic and non-hematologic adverse events graded according to CTCAE version 5.0. Bars represent the percentage of patients experiencing each toxicity by maximum grade. Most adverse events were grade 1–2. Grade ≥3 toxicities were primarily hematologic, including neutropenia, anemia, and thrombocytopenia. No grade 5 events were observed.

The safety profile of platinum rechallenge was consistent with the known toxicity of platinum-based regimens. The most common adverse events (any grade) were fatigue (78%), anemia (73%), neutropenia (63%), nausea/emesis (48%), peripheral neuropathy (43%), alopecia (43%), thrombocytopenia (30%), diarrhea (25%), constipation (22%), anorexia (21%), and hypersensitivity reactions to platinum (21%). Most events were grade 1 or grade 2. The most common grade ≥3 adverse events were neutropenia (43%), anemia (13%), and thrombocytopenia (11%). No grade 5 adverse events were observed, and no adverse events led to treatment discontinuation [Figure 4].

## 4. Discussion

The objective response rate in our cohort demonstrates meaningful clinical activity of platinum rechallenge in selected patients with platinum-resistant disease. Importantly, our cohort represents a heavily pretreated population (median six prior lines) who had achieved an extended platinum-free interval through non-platinum therapies, suggesting that platinum sensitivity can be restored in appropriately selected patients [7,9,10,19].

In patients with *BRCA*-mutated tumors, platinum rechallenge was associated with a higher objective response rate and significantly longer progression-free survival compared with *BRCA* wild-type patients. This finding is biologically plausible and consistent with the central role of BRCA1 and BRCA2 in homologous recombination repair. Platinum compounds induce DNA adducts and intra- and interstrand cross-links that ultimately generate replication-associated double-strand DNA breaks [11]. In tumors with loss of *BRCA* function, homologous recombination repair is impaired, limiting the ability of cancer cells to accurately repair platinum-induced DNA damage and thereby increasing platinum sensitivity [20,21,22]. By contrast, *BRCA* wild-type tumors may retain more proficient homologous recombination repair capacity or may acquire alternative mechanisms of platinum resistance under therapeutic pressure [11,13]. Notably, 38.5% of *BRCA*-mutated patients achieved a platinum-free interval greater than 6 months after rechallenging, a finding that supports potential platinum re-sensitization in selected patients and remains rarely documented in the post-PARP inhibitor era. Although the small number of BRCA-mutated patients (*n* = 13) warrants cautious interpretation, the magnitude and consistency of benefit across response rate, progression-free survival, and post-rechallenge platinum-free interval suggest that BRCA status may serve as a potential predictive biomarker for patient selection in this setting.

The higher proportion of *BRCA*-mutated patients achieving a platinum-free interval greater than 6 months after rechallenge supports the concept that platinum sensitivity may be dynamic rather than fixed [2,7]. Several non-mutually exclusive mechanisms may contribute to this phenomenon. A prolonged period without platinum exposure may reduce selective pressure for platinum-resistant clones and allow residual platinum-sensitive subclones to re-emerge. In addition, intervening non-platinum therapies may alter clonal composition, and tumor evolution may modify DNA repair dependency over time. Lower cumulative treatment pressure may preserve tumor genomic profiles closer to the original platinum-sensitive state, with less clonal evolution, spatial heterogeneity, and selection of resistant subclones [13,19]. Conversely, acquired resistance mechanisms such as *BRCA* reversion mutations, restoration of homologous recombination proficiency, replication fork protection, drug efflux, or selection of resistant subclones may reduce platinum sensitivity, particularly in heavily pretreated patients [19,21,23,24]. These mechanisms may explain why patients with *BRCA*-mutated tumors and fewer prior treatment lines showed numerically more favorable outcomes in our cohort. However, this observation should be interpreted with caution, as the subgroup included very few patients, and the study was not powered to detect definitive interactions between *BRCA* status and prior treatment burden. Therefore, these results should be regarded as hypothesis-generating.

Beyond *BRCA*1/2, additional molecular alterations may also influence platinum rechallenge outcomes [13,25]. In the present study, additional molecular profiling was available for a subset of patients and is summarized descriptively, including alterations in *TP53*, *CCNE1*, *RB1*, *PTEN*, Fanconi pathway genes, and *NF1*/*NF2*. However, these data were heterogeneous in terms of testing platform, gene coverage, tissue timing, and reporting format and were therefore not incorporated into formal efficacy analyses. In our cohort, standardized HRD testing was not available for most patients, as HRD testing was not standard practice during most of the study period. Therefore, the *BRCA* wild-type subgroup should not be interpreted as a homologous recombination-proficient population, as some *BRCA* wild-type tumors may still harbor HRD through alternative mechanisms, including *RAD51C/D* alterations, *BRCA*1 promoter methylation, or other defects in homologous recombination repair. This molecular heterogeneity may have contributed to the responses observed among *BRCA* wild-type patients and limits the generalizability of *BRCA*-stratified outcomes. *RAD51* functional assays were not performed as part of routine clinical practice [26,27,28]. A comprehensive integration of HRD status, *RAD51* functional assessment, *BRCA* reversion mutations, and other mechanisms of platinum resistance would require dedicated molecular reanalysis and represent the basis for a future translational study focused on molecular predictors of platinum rechallenge benefit.

Our findings align with emerging evidence challenging the traditional platinum-resistant paradigm. Recent retrospective studies have reported response rates of 23–55% with platinum rechallenge in patients with platinum-resistant disease who achieved extended platinum-free intervals [9,10]. Most notably, Valenza et al. recently reported an objective response rate of 27% and a median progression-free survival of 5.4 months in 30 patients in the platinum-resistant setting rechallenged with platinum [10]. Our larger cohort with molecular stratification by *BRCA* status demonstrates superior outcomes, likely reflecting more stringent patient selection, including ≥12 months of intervening non-platinum therapy before platinum rechallenge, and excluding platinum-refractory disease. These results contrast with earlier retrospective published series, largely influenced by evolving therapeutic standards, patient selection criteria, treatment sequencing, and response assessment methods [14,15,29].

The platinum rechallenge regimen was not included in the multivariable model because of the limited sample size, the distribution across multiple regimen categories, and the risk of model overfitting. Therefore, comparisons between platinum doublets should be considered exploratory. 

A key strength of our study is the molecular stratification by *BRCA* status—a critical predictive biomarker not evaluated in most prior platinum rechallenge studies [9,10]—suggesting its predictive value for platinum rechallenge outcomes. However, *BRCA* status was not reassessed immediately prior to retreatment, and temporal molecular evolution—including *BRCA* reversion mutations—could alter functional homologous recombination status [19,21,30]. Obtaining fresh tumor biopsies in heavily pretreated patients presents significant practical challenges. Liquid biopsy approaches for monitoring *BRCA* reversion mutations and other resistance mechanisms show promise but remain investigational [24,31]. Future studies should explore additional predictive biomarkers, including functional HRD assays and RAD51 foci formation, which may more accurately identify patients likely to benefit from platinum rechallenge [27,28].

Other key strengths of our study include a contemporary patient cohort (2010–2023), reflecting modern treatment paradigms, including widespread PARP inhibitor use. Notably, one-third of our patients had prior PARP inhibition exposure, which was associated with longer progression-free survival after platinum rechallenge in exploratory analysis [Figure 2]. However, this finding should be interpreted in the context of conflicting data in the literature, with some studies reporting reduced platinum sensitivity immediately post-PARP inhibitor therapy, particularly in *BRCA*-mutated patients [30,32,33]. This association is likely multifactorial and may reflect enrichment for *BRCA*-mutated or HRD tumors, selection of patients with more indolent disease biology, longer prior disease control, or better clinical fitness to receive subsequent platinum. Therefore, this finding should not be interpreted as a causal effect of PARP inhibitors on platinum re-sensitization.

Our findings align with recent ESMO 2023 guidelines, which acknowledge platinum rechallenge as a viable option in patients with platinum-resistant ovarian cancer whose disease did not progress through prior platinum therapy [8]. The real-world nature of our cohort, including heavily pretreated patients (median 6 prior lines), enhances generalizability to contemporary clinical practice.

This study has several limitations, including its retrospective, single-center design and the absence of a control group. Selection bias is inherent, as patients selected for platinum rechallenge are likely to represent a subgroup with more favorable clinical characteristics. The relatively small number of patients with *BRCA*-mutated tumors and the lack of HRD status for most cases limit biomarker-driven interpretation.

The inherent variability in CT assessment intervals in retrospective practice may introduce bias into progression-free survival estimation. In this context, objective response rate provides a complementary and more direct measure of antitumor activity. In addition, the long study period introduces heterogeneity in prior treatments, including increasing use of PARP inhibitors. We did not systematically distinguish progression during versus after PARP inhibitor therapy, which may represent clinically distinct scenarios. Our findings suggest that prior PARP inhibitor exposure does not uniformly preclude benefit from platinum rechallenge. Despite these limitations, our findings provide hypothesis-generating data supporting the role of *BRCA* status in predicting benefit from platinum rechallenge and warrant prospective validation.

This study evaluated the efficacy of platinum-based rechallenge in patients with platinum-resistant ovarian cancer, a population traditionally considered non-candidates for further platinum-based therapy and explored clinical and molecular predictors of benefit. Despite recent therapeutic advances, including mirvetuximab soravtansine, prognosis in the platinum-resistant setting remains poor, with most phase III trials failing to meet their primary endpoints [5]. Treatment options are limited, and no validated biomarker currently guides therapy selection beyond FRα expression in patients with no more than three prior treatment lines [4]. In the current therapeutic landscape, where antibody–drug conjugates are emerging, identifying patients who may still benefit from platinum rechallenge remains clinically relevant.

Our findings suggest that platinum rechallenge may represent a clinically meaningful therapeutic option in carefully selected patients, supporting a shift from a rigid resistance-based paradigm toward a more dynamic, biology-driven approach to treatment sequencing in clinical practice. These results also identify *BRCA* mutation status and prior treatment burden as key factors to be considered when evaluating platinum rechallenge strategies and support their prospective assessment in future research.

## 5. Conclusions

Platinum rechallenge showed clinically meaningful antitumor activity in a selected subgroup of patients with platinum-resistant ovarian cancer who had received an extended interval of intervening non-platinum therapy. The benefit appeared greatest among patients with *BRCA*-mutated tumors and lower prior treatment burden, supporting the concept that platinum resistance is not always irreversible and may be modulated by tumor biology and treatment sequencing. These findings suggest that *BRCA* status, together with clinical selection factors such as prolonged interval since prior platinum treatment and number of prior treatment lines, may help identify patients for whom platinum rechallenge remains a reasonable therapeutic option. Given the retrospective design, limited sample size, absence of a control group, and incomplete standardized molecular profiling, these results should be considered hypothesis-generating and require prospective validation with integrated genomic and functional HRD biomarkers.

## Figures and Tables

**Table 1:** Baseline clinicopathologic characteristics of patients undergoing platinum rechallenge, overall and according to *BRCA* mutation status.

	Overall Population *n* = 63	*BRCA* WT *n* = 46	*BRCA* Mut *n* = 13	*p*-Value
**Age** **in years, median (IQR)**	57 (47–63)	59 (49–64)	51 (44–62)	0.102
**Performance status:**				0.766
ECOG 0	24 (40%)	18 (41%)	6 (50%)	
ECOG 1	34 (57%)	25 (57%)	6 (50%)	
ECOG 2	2 (3%)	1 (2%)	0 (0%)	
Missing data, n	3	-	-	
**Histological subtype:**				0.418
High-grade serous	57 (92%)	41 (91%)	12 (92%)	
High-grade endometrioid	2 (3%)	1 (2%)	1 (8%)	
Clear cells	3 (5%)	3 (7%)	0 (0%)	
Missing	1	-	-	
**Primary platinum resistance,** ***n*** **(%)**	11 (17%)	11 (24%)	0 (0%)	0.121
**Number of prior platinum lines:**				0.024
1	7 (11%)	5 (11%)	1 (8%)	
2	33 (52%)	26 (57%)	6 (46%)	
3	15 (24%)	12 (26%)	1 (8%)	
4	8 (13%)	3 (7%)	5 (38%)	
**Prior lines, median (IQR)**	6 (5–6)	6 (5–6)	5 (4–6)	0.444
**Platinum-free interval before platinum rechallenge in months, median (IQR)**	25 (20–33)	25 (19–34)	21 (15–25)	0.051
**Platinum rechallenge regimen:**				0.338
Carboplatin–paclitaxel	24 (38%)	16 (35%)	4 (31%)	
Carboplatin–pegylated liposomal doxorubicin	25 (40%)	17 (37%)	8 (62%)	
Carboplatin–gemcitabine	12 (19%)	11 (24%)	1 (8%)	
Carboplatin monotherapy	2 (3%)	2 (4%)	0	
***BRCA*** **status:**				
t*BRCA*1	11 (17%)	-	-	-
t*BRCA*2	2 (3%)	-	-	-
t*BRCA* wild type	46 (73%)	-	-	-
Unknown	4 (6%)	-	-	-

Data are presented as *n* (%) unless otherwise indicated. IQR = interquartile range; ECOG = Eastern Cooperative Oncology Group performance status.

**Table 2:** Descriptive summary of additional molecular alterations among patients with available molecular profiling.

Gene Alteration	Patients with Alterations, *n*	Pathogenic/Likely Pathogenic Variant	CN Loss/LoF	CN Gain/Amplification
***TP53***	32	32	0	0
***CCNE1***	4	0	0	4
***RB1***	3	2	0	1
***PTEN***	3	3	0	0
***FANCL***	2	2	0	0
***FANCA***	1	0	1	0
***NF1***	1	0	1	0
***NF2***	2	0	1	1
***CDKN2A***	2	2	0	0
***CDK12***	2	1	0	1
***ARID1A***	1	1	0	0
***ARID1B***	1	0	1	0
***NOTCH***	3	1	0	2
***PIK3CA***	1	1	0	0
***KRAS***	1	1	0	0
***BRIP1***	1	1	0	0
RAD51C *	1	1	0	0
RAD51D *	1	1	0	0
***ERBB2***	1	0	0	1

Data are shown among the 40 patients with available additional molecular profiling beyond *BRCA*1/2. Categories are not mutually exclusive when a gene has more than one type of alteration across different patients. CN = copy number; LoF = loss of function. CN loss/LoF was assigned only when the molecular report explicitly described loss, deletion, loss of function, or equivalent wording. CN gain/amplification includes reported gain, amplification, or duplication. NOTCH includes alterations in NOTCH family genes. * RAD51C and RAD51D pathogenic/likely pathogenic variants were detected as germline alterations in the same patient.

**Table 3:** Response outcomes following platinum rechallenge in the overall cohort and stratified by *BRCA* mutation status.

	Global (%) *n* = 63	*BRCA* Mut *n* = 13	*BRCA* WT *n* = 46	*p*-Value
**ORR**	36 (57.1%)	10 (76.9%)	23 (50.0%)	0.159
**CR**	2 (3.2%)	1 (7.7%)	1 (2.2%)	0.362
**PR**	34 (54.0%)	9 (69.2%)	22 (47.8%)	0.294
**SD**	17 (27.0%)	0 (0%)	16 (34.8%)	0.033
**DCR**	53 (84.1%)	10 (76.9%)	39 (84.8%)	0.804
**PD**	10 (15.9%)	3 (23%)	7 (15.2%)	0.804
**TFIp ≥ 6 m**	11 (17.5%)	5 (38.5%)	6 (13.0%)	0.094

ORR = objective response rate; CR = complete response; PR = partial response; SD = stable disease; PD = progressive disease; DCR = disease control rate; TFIp = treatment-free interval of platinum ≥6 months; mut = mutated; WT = wild type.

## Data Availability

The data underlying this study are available from the corresponding author upon reasonable request, subject to institutional and ethical restrictions related to patient confidentiality.

## Supplementary Materials

The following supporting information can be downloaded at https://www.mdpi.com/article/10.3390/cancers18132062/s1; Figure S1: Kaplan–Meier curves for overall survival (OS). (**A**) Entire cohort. (**B**) Stratified by number of prior treatment lines (≤4 vs. >4). (**C**) Stratified by BRCA mutation status. (**D**) Combined stratification by BRCA status and prior treatment lines. Median OS and hazard ratios (HR) are shown where applicable. Dashed horizontal and vertical lines denote the 50% survival probability and the corresponding median progression-free survival estimate, respectively.
