# Supplementary material for: Platinum Rechallenge in Platinum-Resistant Ovarian Cancer: Clinical Outcomes and the Impact of BRCA Status
Source: Cancers (Basel). 2026 Jun 25;18(13):2062. doi: 10.3390/cancers18132062 (PMC13359466; doi:10.3390/cancers18132062)
Supplement: Supplementary file 1 [file cancers-18-02062-s001.zip › cancers-4330265-supplementary.pdf]

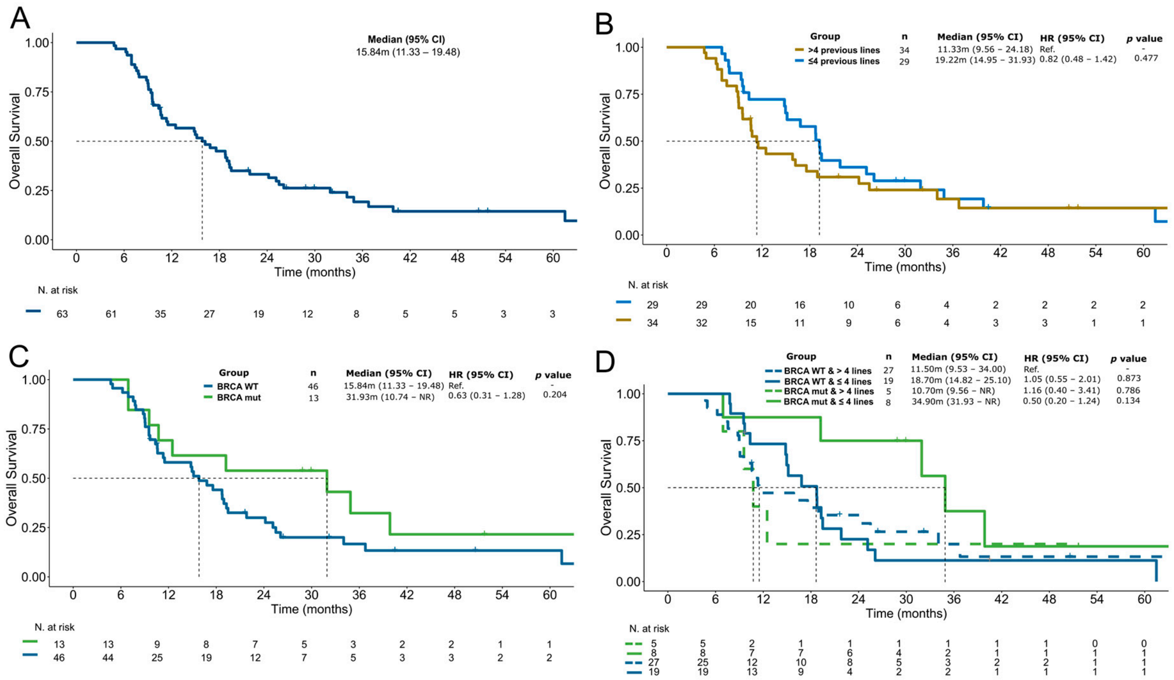

Figure S1. Kaplan–Meier curves for overall survival (OS). (**A**) Entire cohort. (**B**) Stratified by number of prior treatment lines (≤4 vs >4). (**C**) Stratified by BRCA mutation status. (**D**) Combined stratification by BRCA status and prior treatment lines. Median OS and hazard ratios (HR) are shown where applicable. Dashed horizontal and vertical lines denote the 50% survival probability and the corresponding median progression-free survival estimate, respectively.
